# Perovskite Photocatalytic CO_2_ Reduction or Photoredox Organic Transformation?

**DOI:** 10.1002/anie.202205572

**Published:** 2022-08-23

**Authors:** Jovan San Martin, Nhu Dang, Emily Raulerson, Matthew C. Beard, Joseph Hartenberger, Yong Yan

**Affiliations:** ^1^ Department of Chemistry and Biochemistry San Diego State University San Diego CA 92182 USA; ^2^ National Renewable Energy Laboratory Golden CO 80401 USA; ^3^ Renewable and Sustainable Energy Institute University of Colorado Boulder CO 80309 USA

**Keywords:** CO Surrogate, CO_2_ Reduction, Perovskite, Photocatalytic Organic Reactions, Photoredox

## Abstract

Metal‐halide perovskites have been explored as photocatalysts for CO_2_ reduction. We report that perovskite photocatalytic CO_2_ reduction in organic solvents is likely problematic. Instead, the detected products (i.e., CO) likely result from a photoredox organic transformation involving the solvent. Our observations have been validated using isotopic labeling experiments, band energy analysis, and new control experiments. We designed a typical perovskite photocatalytic setup in organic solvents that led to CO production of up to ≈1000 μmol g^−1^ h^−1^. CO_2_ reduction in organic solvents must be studied with extra care because photoredox organic transformations can produce orders of magnitude higher rate of CO or CH_4_ than is typical for CO_2_ reduction routes. Though CO_2_ reduction is not likely to occur, in situ CO generation is extremely fast. Hence a suitable system can be established for challenging organic reactions that use CO as a feedstock but exploit the solvent as a CO surrogate.

## Graphical abstract

Halide perovskite photocatalytic CO_2_ reduction in organic solvent is likely problematic. The observed CO product clearly results from photocatalytic organic transformation involving solvents (i.e., ethyl acetate or chloroform). Perovskite‐photocatalyzed generation of CO from solvent at a fast rate may create synthetic opportunities for useful organic syntheses using organic solvent as the CO surrogate.

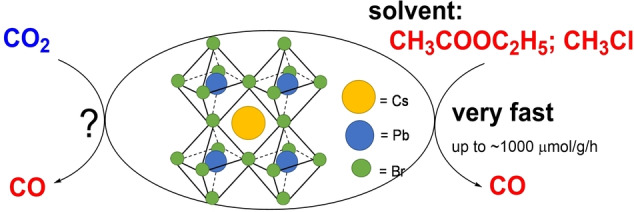

## Introduction

Utilization of renewable energy for the efficient conversion of deleterious CO_2_ to value‐added fuels is a green solution that addresses challenges in both energy demand and environmental concern.[^1^, ^2^, ^3^, ^4^, ^5^] As a result, ongoing research efforts are developing methods to capture renewable solar energy for highly efficient CO_2_ conversion. Suitable and efficient photocatalysts can play an essential role in this goal by addressing two key problems: 1) highly efficient solar energy capture, and 2) highly active chemical transformations from inert CO_2_ to value‐added fuels or chemicals.[^2^, ^6^, ^7^] Numerous research efforts have been conducted for photocatalytic CO_2_ reduction and the catalysts involved in such efforts include, but are not limited to, transition metal complexes,^[8]^ metal‐organic frameworks,^[9]^ polymeric chromophores,^[10]^ and various semiconductors.^[11]^

Lately, lead halide perovskite semiconductors have shown exceptional ability to capture and transform solar energy to electricity. In addition, a few examples demonstrate that lead halide perovskite nanocrystals (NCs) are efficient and useful photocatalysts for various reactions,[^12^, ^13^, ^14^, ^15^] including the hydrogen evolution reaction (HER),^[16]^ organic transformations,[^17^, ^18^, ^19^, ^20^, ^21^] and photocatalytic CO_2_ reduction.[^22^, ^23^] Unique optoelectronic properties that make perovskite good solar cells are also important when designing robust and highly efficient photo‐catalysts, including the photoinduced CO_2_ reduction systems.[^12^, ^14^] Two pioneer reports demonstrated perovskite NCs in such photocatalytic CO_2_ reduction reactions.[^22^, ^23^] Thereafter, enhanced performance of CO_2_ reduction to CO or CH_4_ have been extensively explored using perovskite NCs incorporated into various catalytic reactor systems. These research efforts have focused on constructing various hybrid interfaces in order to efficiently separate the photoexcited electron‐hole pairs and then employ them in photocatalysis.[^12^, ^14^] Such efforts include but are not limited to heterostructures of, CsPbBr_3_/graphene oxide,^[23]^ CsP‐bBr_3_/g‐C_3_N_4_,^[24]^ CsPbBr_3_@TiO‐CN,^[25]^ CsPbBr_3_/MXene,^[26]^ CsPbBr_3_/BZNW/MRGO,^[27]^ CsPbBr_3_ NCs/Pd,^[28]^ CsPbBr_3_‐Re(600),^[29]^ CsPbBr_3_/TiO_2_,^[30]^ CsPbBr_3_@ZIF‐67,^[31]^ CsPbBr_3_/UiO_66_(NH_2_),^[32]^ CsPbBr_3_/Fe,^[33]^ CsPbBr_3_/Cs_4_PbBr_6_ NCs/Co,^[34]^ CsPbBr_3_ NC/Mn,^[35]^ MAPbI_3_@PCN‐221,^[36]^ CH_3_NH_3_PbBr_3_/GO,^[37]^ MAPbI_3_/ In_0.4_Bi_0.6_,^[38]^ as well as lead‐free perovskite systems, such as, Cs_3_Bi_2_I_9_ NCs,^[39]^ Cs_2_SnI_6_/SnS_2_ NCs,^[40]^ Cs_2_AgBiBr_6_ NCs^[41]^ etc.

Considering the instability of perovskite NCs in polar solvents or aqueous conditions, the reported photocatalytic CO_2_ reductions employ less polar organic solvents, most commonly ethyl acetate.^[12]^ The use of these low polarity solvents has been justified in order to ensure a long‐term stability and simultaneously a high CO_2_ solubility.^[12]^ The proposed mechanism utilizes photoexcited electrons to reduce CO_2_ to CO or CH_4_, while photoexcited holes oxidize water and generate O_2_,^[22]^ overall leading to a CO_2_ transformation to fuels with solar light as energy input and water as an intermediate [Eqs. (1)–3].

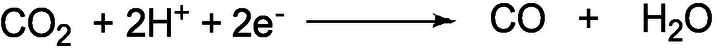




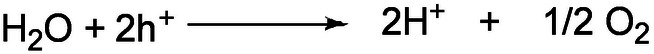




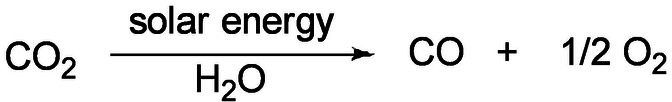




However, an intrinsic problem that can occur when using organic solvents is the potential for direct photolysis of the solvent,; i.e., ethyl acetate under ultraviolet illumination. Such side‐reactions can also yield CO and alkane products.[^42^, ^43^] Furthermore, a recent report did not detect O_2_ produced from the water oxidation half reaction [Eq. (2)] and questioned if the carbonaceous product actually originated from CO_2_ reduction under liquid‐phase photocatalysis and full arc conditions.^[44]^ Here we show that under visible‐light illumination (i.e., 456 nm LED), mediation of photocatalytic CO_2_ reduction over CsPbBr_3_ NCs in typical organic solvent is not likely to occur. Instead, we find that the observed CO or CH_4_ products result from a photocatalytic organic transformation involving the organic solvent.

## Results and Discussion

^**13**^ **C‐Labeling experiments** represent the most reliable validation of CO_2_ reduction. Under the typical photocatalysis setup^[12]^ (i.e., ethyl acetate as the organic solvent and colloidal CsPbBr_3_ NCs ≈10 nm in size^[19]^ as the catalyst; details in the Supporting Information), we have successfully observed both CO and CH_4_ as products under blue LED illumination. We found a rate of CO production (*R*_CO_) of ≈6 μmol g^−1^ h^−1^ and ≈1 μmol g^−1^ h^−1^ for CH_4_ (*R* ${}_{\mathrm{CH}{}_{4}}$ ). The overall average electron yield was ≈20 μmol g^−1^ h^−1^ (*R*_electron_) according to the equation (*R*_electron_=2 *R*_CO_+8 *R* ${}_{\mathrm{CH}{}_{4}}$ +2 *R* ${}_{H{}_{2}}$ ). This result corroborates and confirms the initial observation of CO and CH_4_ generation.[^22^, ^23^]

However, to our surprise the ^13^C‐labeling experiments suggest a different reaction mechanism (Table 1). We found that, even when ^13^CO_2_ is dissolved in ethyl acetate, the product consists of roughly the natural abundance of ^12^CO (Table 1, entry b). Our finding contrasts with ^13^C‐labeled CO_2_ reduction reports. For instance, some literature reports indicate an almost 100 % ^13^CO was generated from ^13^CO_2_ using CsPbBr_3_ NCs.[^22^, ^25^, ^29^, ^34^, ^36^] But these reports are confusing; for instance, it was claimed that “under Ar atmosphere, a certain amount of CO and CO_2_ was obtained, indicating partial photo‐oxidation of ethyl acetate.”^[22]^ If the ethyl acetate (non‐labeled) photo‐conversion to CO is observed and such a route is truly unavoidable, as shown from many reports, there must be a source of non‐labeled CO that originates from the solvent. It is confusing to observe the isotope‐labeling experiment with ^13^CO_2_ but also show the same abundance, roughly ≈100 atom % of ^13^CO or ^13^CH_4_ products (i.e., essentially no ^12^C‐product). Such a claim is apparently contradictory. It is also important to note that the organic solvent photolysis to produce CO that was reported in the 1950 s[^42^, ^43^] and more recently is non‐trivial.^[44]^ In particular the reaction requires high‐intensity UV‐containing illumination; i.e., a 300 W Xe lamp fitted with a standard AM 1.5 G filter.

**Table 1:** Labeling experiments.^[a]^

Entry	Gas	Solvent	CO	CH_4_	Comments
a	CO_2_	CH_3_COOCH_2_CH_3_	^12^CO	^12^CH_4_	^12^C products at natural abundance
b	^13^CO_2_	CH_3_COOCH_2_CH_3_	^12^CO	^12^CH_4_	99 atom % ^13^CO_2_ but nearly natural abundance products
c	CO_2_	CH_3_^13^COOCH_2_CH_3_	^13^CO	^12^CH_4_	^13^CO (52 %) and nearly natural abundance ^12^CH_4_
d	CO_2_	^13^CH_3_^13^COOCH_2_CH_3_	^13^CO	^13^CH_4_	^13^CO (61 %) ^13^CH_4_ (27 %)
e	CO_2_	^13^CH_3_^13^COO^13^CH_2_C^13^H_3_	^13^CO	^13^CH_4_	^13^CO (97 %) ^13^CH_4_ (97 %)
f	air	^13^CH_3_^13^COO^13^CH_2_C^13^H_3_	^13^CO	^13^CH_4_	^13^CO (97 %) ^13^CH_4_ (97 %)
g	O_2_	^13^CH_3_^13^COO^13^CH_2_C^13^H_3_	^13^CO	^13^CH_4_	^13^CO (97 %) ^13^CH_4_ (97 %)

[a] Conditions: ethyl acetate (1 mL) with CsPbBr_3_ nanocrystals (1 mg), saturated with respective gas and sealed with septum, illuminated under 456 nm LED Kessil LED. Headspace was detected by GCMS. ^13^C‐label using 99 atom%.

While most perovskite photocatalytic reduction studies have not employed isotopic labeling, we analyzed past reports that did use ^13^CO_2_‐labeled feedstock and the comparison has been summarized in Table S1 (Supporting Information). If the produced CO and CH_4_ originate from CO_2_, their atom% label should be the same and both near the same level as the CO_2_ feedstock, but we found that the atom% of ^13^CO and ^13^CH_4_ are not the same in these reports.[^28^, ^30^, ^31^]

These results triggered us to re‐examine the photocatalytic process, particularly under visible‐light illumination, and trace the source of CO and CH_4_ products. We found that, under our conditions, the CO or CH_4_ products are clearly sourced from ethyl acetate (see Table 1). Entries c and d in Table 1 indicate that the CO is mainly from the 1‐C (see structure in entry c) of ethyl acetate with a minor contribution from 2‐C (see structure in entry d) or other carbon atoms of the ethyl acetate. The CH_4_ product is most likely sourced from the 2‐, 3‐, or 4‐C position of the ethyl acetate. Entry e clearly indicates that neither the carbon in CO nor CH_4_ has been sourced from the CO_2_ feedstock, indicating a minimum amount of CO_2_ reduction to CO or CH_4_ under our synthetic conditions. A minor atom% decrease from 99 % in ethyl acetate ^13^C to 97 % in CO or CH_4_ is perhaps either from the experimental error or from the other organic residue in this solvent, or luckily from CO_2_ reduction, if there is any. In any case, CO_2_ cannot be the major source responsible for the observed CO or CH_4_.

If the CO_2_ reduction is not the main source for the observed CO or CH_4_, there should be no need to have CO_2_ present in order to detect those products. We find that under the ^13^C‐labled ethyl acetate condition and without CO_2_ (i.e., just air‐ or oxygen‐saturated solvents; Table 1, entry f or g), the observation at the GCMS level shows no difference with entry e which contained solvents saturated with CO_2_. All these experiments clearly indicated that 1) CO_2_ should be at least not the major source for the CO or CH_4_ products, and 2) the observed products mainly originated from a photocatalytic organic transformation under visible light.

**Figure 1:**
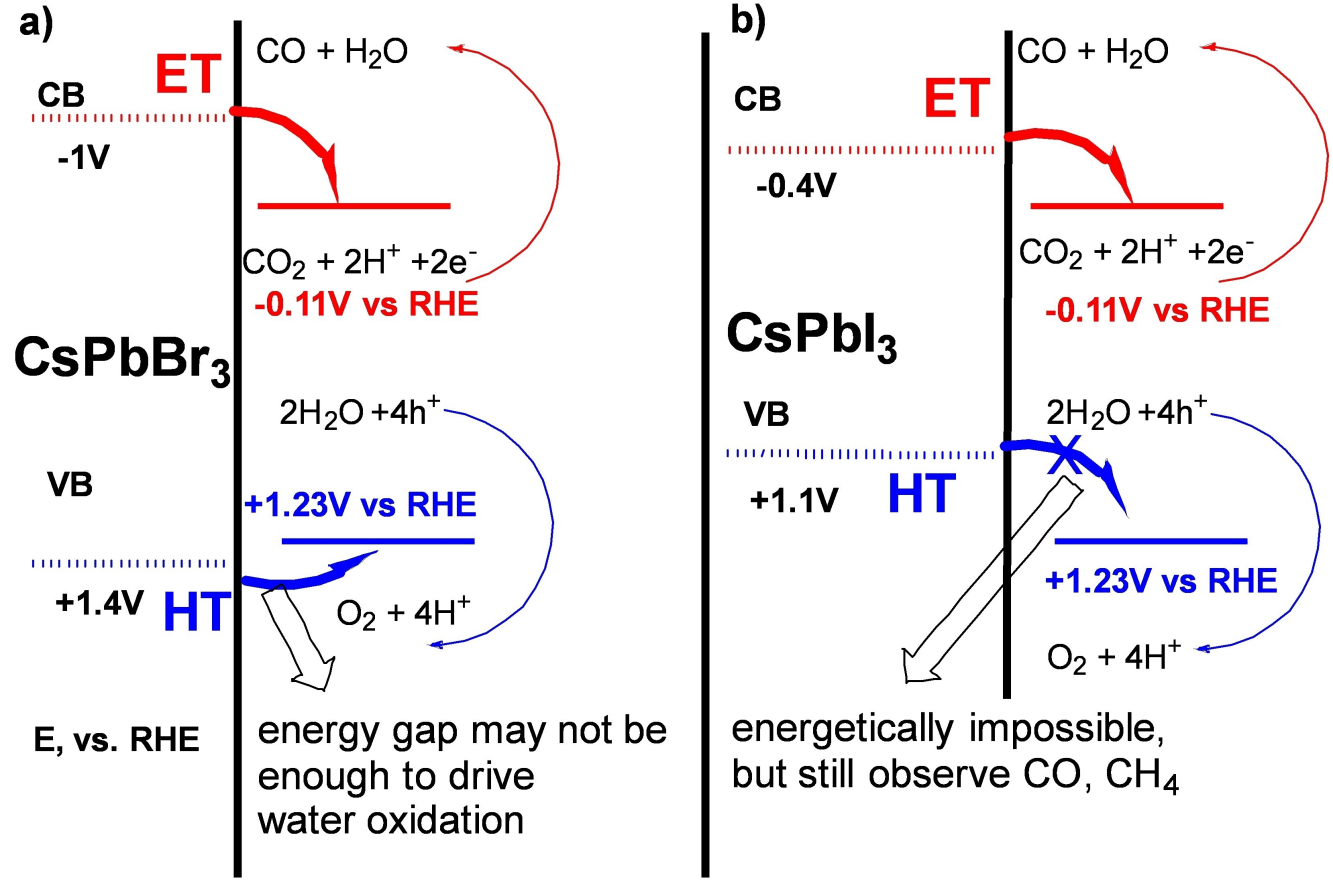
Band energy comparison with half‐cell reactions: a) CsPbBr_3_, b) CsPbI_3_.[^14^, ^15^] (CB, VB energy according to references).[^14^, ^15^]

**Band energy analysis**: The perovskite photocatalytic CO_2_ reduction has been proposed to proceed via photoexcited charge carriers; i.e., electrons from the conduction band (CB) reduce the CO_2_, listed as photocathode reaction [Eq. (1)], and holes from the valence band oxidize water (i.e., the other half‐cell reaction is a photoanode reaction [Eq. (2)].[^22^, ^23^] First, from a band energy level perspective, we question whether there is enough thermodynamic driving force to induce such charge‐carrier transfer reactions. The energy level (Figure 1) for the electron transfer from the CB is −1 V vs. RHE, and thus is sufficiently more negative than the reduction potential needed to drive the reduction reaction [Eq. (1)].^[12]^

However, it is indeed problematic from an energetic standpoint to be able to drive the photoanode half‐cell reaction [Eq. (2)]. Unless the system is able to harvest hot carriers to drive the oxidation; i.e., the driving force from the valence band edge may not be sufficient to induce water oxidation. Four holes are involved in the water oxidation reaction and as a result the activation barrier is generally found to be quite high. In fact, the state‐of‐the‐art water oxidation catalyst requires at least 0.3 V (i.e., IrOx) of overpotential.^[45]^ Unless CsPbBr_3_ NCs are extremely active toward water oxidation, the 0.17 V of excess energy difference (Figure 1) may not be sufficient to drive the Equation (2), supporting the previous report of unsuccessful detection of O_2_ from the similar photocatalytic setup.^[44]^

Furthermore, we have tuned the band energy of perovskite using an iodide anion‐exchange reaction that reduces the band gap of the NCs, or directly using CsPbI_3_ NCs to intentionally move the VB higher, reaching up to +1.1 V vs. RHE, which is ≈0.1 V less positive than the potential needed to drive the water oxidation half‐cell reactions (i.e., +1.23 V vs. RHE; Figure 1b). In this case we should not be able to drive the CO_2_ reduction reaction [Eq. (3)]; however, we find that under the same photocatalytic conditions a significant amount of CO and CH_4_ can still be detected (details in the Supporting Information), indicating that the photoanode reaction Equation (2) is likely not necessary for CO or CH_4_ generation.

Our photocatalytic system, as noted above, is homogeneously dispersed in the solvent but does present a heterogeneous NC surface and that surface can present opportunities to affect reactions. For example, semiconductor band bending in respective solvent, substrate‐surface binding, defects and/or surface trap states may alter the VB energy level to make Equation (2) possible. Furthermore, hot‐carrier‐based charge transfer may also permit such hole‐transfer reaction for water oxidations.[^46^, ^47^] In general, heterogeneous photocatalysis heavily depends on the surface properties and it is not always true to estimate the electron and hole transfer probabilities purely based upon CB and VB values.^[48]^ For instance, the surface properties on the gas‐solid interface might be completely different than that of the organic‐solid surface for CO_2_ reduction.^[44]^ However, under our photocatalytic reaction conditions, as discussed above, we found that eq. 2 is not necessary for CO generation. Here we have carefully processed the organic solvent (i.e., to obtain a water‐free ethyl acetate). In fact, we find the generation of CO and CH_4_ using the water‐free solvent demonstrated a slightly higher CO generation rate compared to a system with 0.3 % v/v water/ethyl acetate. Thus, the intentional addition of water into the solvent system plays little effect in the product formation. Our observation is in line with the initial report of perovskite NC photocatalytic CO_2_ reduction in which water is not involved in the reaction.^[23]^ Other reports that do not include water in the reaction can still find CO and CH_4_ products.^[12]^

**The role of oxygen**: Almost all reports for perovskite photocatalytic CO_2_ reduction have conducted control experiments. The main control compares the photocatalytic product under an inert gas (N_2_ or Ar) sparging to that when CO_2_ is bubbled through the system.[^22^, ^23^] Such control experiments are indeed reproduced in our lab in which we find a much higher CO production rate under CO_2_ conditions than under N_2_ or Ar sparging (Table 2, entries a–c).

**Table 2:** Control experiment.^[a]^

Entry	gas	CsPbBr_3_	CO	CH_4_	Comments
a	CO_2_	1 mg	5	1	Repeatable
b	N_2_	1 mg	<0.1	<0.1	Repeatable
c	Ar	1 mg	<0.1	<0.1	Repeatable
d	air	1 mg	24	4	New Control
e	CO_2_	0	<0.1	<0.1	Repeatable
f	air	0	<0.1	<0.1	New Control
g	O_2_	1 mg	151	27	New Control

[a] Rate: μmol g^−1^ h^−1^.

However, we suspect that such control experiments cannot absolutely exclude CO or CH_4_ originating from an organic transformation of the solvent. To demonstrate, we conducted new control experiments and the results are summarized in Table 2. Surprisingly, we find that air‐saturated ethyl acetate (Table 2, entry d) generates 400 % higher amounts of CO than does the CO_2_‐saturated solution under the same photocatalytic conditions. Therefore, CO_2_ is not a necessary component. Rather it is the presence of oxygen that plays an important and necessary role in production of CO. In fact, Table 2, entry g, details the case for pure oxygen sparging and leads to an over 3 000 % increase in the CO production rate. Thus, the oxygen's partial pressure impacts the CO and CH_4_ generation. This result also agrees with our labeled experiment.

Our control experiment indicates a role for O_2_ in the generation of CO or CH_4_ in our system. We further explored the role of oxygen and the results are summarized in Table 3. For instance, entries b–d, when O_2_ concentration is fixed at ca. 80 % with gas sparging controlled by two separate gas flow meters, gives an identical CO production rate or ≈125 μmol g^−1^ h^−1^ regardless if the O_2_ is mixed with N_2_, Ar, or CO_2_. The production rate only changes significantly if the O_2_ concentration is altered (e.g., ≈78 μmol g^−1^ h^−1^) as shown in entries 5–7 with 50 % O_2_, or ≈25 μmol g^−1^ h^−1^ in entries 8–10 with 20 % O_2_. We find that the partial pressure of O_2_ determines the CO and CH_4_ generation rate, while the other components of the mixed gas, either N_2_, CO_2_, or Ar are essentially non‐distinguishable for the photocatalytic results.

**Table 3:** O_2_ impact on CO and CH_4_.^[a]^

Entry	O_2_%	Mixed gas	CO	CH_4_
a	100 %	Pure oxygen	151	27
b	80 %	Flow rate control, O_2_/N_2_, ca 80 : 20	124	22
c	80 %	Flow rate control, O_2_/CO_2_, ca 80 : 20	123	24
d	80 %	Flow rate control, O_2_/Ar, ca 80 : 20	126	24
e	50 %	Flow rate control, O_2_/N_2_, ca 50 : 50	78	18
f	50 %	Flow rate control, O_2_/CO_2_, ca 50 : 50	78	19
g	50 %	Flow rate control, O_2_/Ar, ca 50 : 50	76	17
h	20 %	Air, O_2_/N_2_, ca 20 : 80	24	4
i	20 %	Flow rate control, O_2_/ CO_2_, ca 20 : 80	24	5
j	20 %	Flow rate control, O_2_/ CO_2_, ca 20 : 80	26	5
k	0.1 %	CO_2_ gas sparging, O_2_ residue read out from O_2_ sensor	6	1
l	<1 ppm	N_2_ gas sparging, O_2_ residue read out from O_2_ sensor	<0.1	<0.1
m	<1 ppm	Ar gas sparging, O_2_ residue read out from O_2_ sensor	<0.1	<0.1
n	<1 ppm	Ultra‐pure CO_2_ gas sparging, O_2_ residue read out from O_2_ sensor	<0.1	<0.1

[a] Rate: μmol g^−1^ h^−1^.

Our results demonstrate that CO_2_ is not necessary for the observation of CO and CH_4_ and, in fact, is essentially inert for the photocatalytic process. The question now is: why does the control experiment delivered by most literature reports and confirmed by us (Table 2, entries a–c), demonstrate a clear difference when the sparging gas is CO_2_ compared to N_2_. We further investigated this case and summarize our results in Table 3, entries k–n. We employed a highly sensitive oxygen sensor to directly read out O_2_ residue after the respective gas sparging. We found that O_2_ residue (headspace readout from O_2_ sensor, ≈1000 ppm or 0.1 %) after CO_2_ sparging is significantly higher than that of N_2_‐ or Ar‐gas sparging (both less than 1 ppm) under our sparging conditions. This is likely because our CO_2_ gas tank contains more O_2_ residue than does the N_2_ or Ar tank. This residual oxygen can impact the outcome of the photocatalytic CO and CH_4_ generation, as we demonstrated above. Another possible reason for the previous failed control experiment is that there might be CO or CH_4_ residue directly from the CO_2_ tank. To further prove this assumption, we employed an ultra‐pure CO_2_ (99.9995 %) gas source to sparge for comparison (Table 3, entry n). With the ultra‐pure CO_2_, we can successfully sparge the system with O_2_ residue in a headspace lower than 1 ppm level, and correspondingly, we do not observe any meaningful CO or CH_4_ generation.

**Figure 2:**
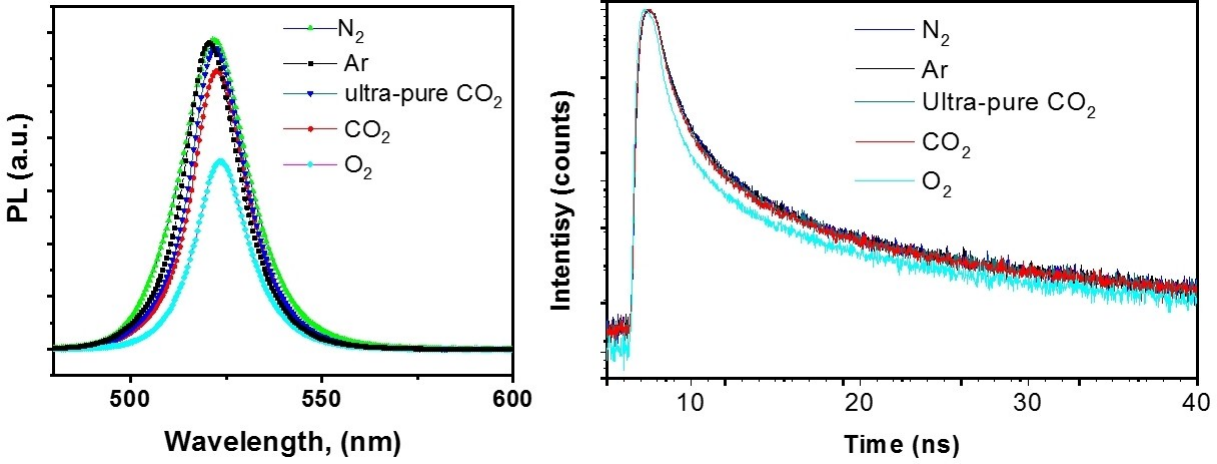
PL intensity and lifetime quenching under different gas‐sparging environments.

**Photoluminescence (PL) quenching experiments** also indirectly prove that CO_2_ is not involved in the photocatalytic reaction. We have conducted gas‐based PL quenching experiments as well as PL lifetime quenching studies (Figure 2). We found that pure N_2_‐ or Ar‐sparging solution show about the same level of PL from CsPbBr_3_ NCs. The ultra‐pure CO_2_‐sparged solutions show no PL difference compared to N_2_ and Ar cases. This result implies that charge or energy transfer between CsPbBr_3_ NCs and CO_2_ does not occur at appreciable levels. While the O_2_‐sparged system shows a moderate and well‐observed PL intensity quenching. PL lifetime measurement also supports this conclusion, showing a decreased PL lifetime when using an O_2_‐saturated solution. It is interesting to note that common grade CO_2_ sparging does show a small amount of PL quenching in both intensity and lifetime. Such quenching is near the edge of experimental error, which we attribute to the oxygen residue from the CO_2_ tank (Table 3, entry k), rather than the CO_2_ molecules themselves.

**Photocatalytic organic transformation**: If CO_2_ is not the source for the observed CO and CH_4_, the carbonaceous products should thus come either from organic residue during the synthesis of the NCs or directly from the organic solvent. We discussed the energy level concerns. Our above control and label experiment indicate that CO_2_ reduction likely does not occur in an organic solvent system. The lack of CO_2_ reduction may be attributed to one or more of the following: the surface of CsPbBr_3_ NCs may be 1) not active enough towards CO_2_ activation under our conditions; 2) more active towards oxygen activation (i.e., electron or energy transfer to O_2_, ultimately leading to photo‐oxidation of organic solvent); 3) more active towards organic matter (i.e., massive amount of organic solvent) than the inert CO_2_. The CB being more negative than the half‐cell reaction [Eq. (1)] or other CO_2_ reducing half‐cell reactions^[12]^ does not necessarily guarantee CO_2_ activation. Kinetics and selectivity may provide a more important role in the current system.

We first explored the possibility of CO generation from the organic capping ligand used to synthesize the NCs. The CsPbBr_3_ NCs employed in photocatalytic studies usually contain surface capping components, most commonly oleic acid, oleylamine etc.^[49]^ These organic capping ligands are directly in contact with the NC surfaces, and hence, can undergo facile charge or energy transfer that could result in CO production. To rule this out, we employed NCs without the use of capping ligands. All inorganic CsPbBr_3_ was synthesized by grinding CsBr and PbBr_2_ (details in the Supporting Information) and the resulting perovskite was employed in the photocatalytic experiment. Surprisingly the non‐terminated perovskite demonstrated an even higher CO and CH_4_ generation rate than NCs (or quantum dots) with capping ligands (Table 4). Simply grinding perovskite generates CO at the rate of 189 μmol g^−1^ h^−1^ under O_2_‐saturated condition, and 30 μmol g^−1^ h^−1^ for air‐saturated conditions. Similarly, such perovskite does not reduce CO_2_ when using ultra‐pure CO_2_ as the sparging gas. Control experiments indicate that employing CsBr or PbBr_2_ by themselves does not lead to any detectable CO or CH_4_. This result excludes the CO or CH_4_ from the organic capping ligand of CsPbBr_3_ NCs. This result also indicates that simple bulk inorganic CsPbBr_3_ is quite active towards photocatalytic CO and CH_4_ generation under visible light.

**Table 4:** Bulk grinding perovskite for CO and CH_4_.

Entry	Catalyst	Gas	CO	CH_4_
a	CsPbBr_3_ grinding from CsBr and PbBr_2_	O_2_	189	32
b	CsPbBr_3_ NC with capping ligand	O_2_	151	27
c	CsPbBr_3_ grinding from CsBr and PbBr_2_	air	30	6
d	CsPbBr_3_ NC with capping ligand	air	24	4
e	CsPbBr_3_ grinding from CsBr and PbBr_2_	Ultra‐pure CO_2_	<0.1	<0.1
f	CsPbBr_3_ NC with capping ligand	Ultra‐pure CO_2_	<0.1	<0.1
g	CsBr only	air	<0.1	<0.1
h	PbBr_2_ only	air	<0.1	<0.1

Metal‐halide perovskite NCs as a photocatalyst is not suitable in high polar solvents (e.g., under aqueous condition) but can work in less polar organic solvents. Such organic solvents provide an overwhelmingly abundant carbonaceous source for the observed CO and CH_4_ products. Organic solvents, particularly acetonitrile, triethanolamine, trimethylamine, and ethyl acetate have been systematically assessed to employ in photocatalytic CO_2_ reduction.^[44]^ Photolysis of many solvents, including the most used ethyl acetate, have been observed to generate CO or CH_4_ under ultraviolet light. Such reports date back to the 1950s and are widely confirmed.^[42]^ We did not detect the ethylene or other alkanes under a visible‐light photocatalytic setup, even though previous photolysis experiments conducted with ultraviolet light did observe those products.^[44]^ Here photocatalytic CO or CH_4_ generation reaction is correlated to O_2_ and ethyl acetate and the CO rate is usually much higher, at least 5 times, than the CH_4_ rate. Therefore, visible‐light‐induced photo‐oxidation of ethyl acetate with perovskite as the photocatalyst is likely to occur in our system and the mechanism is proposed as follows [Eq. 4].

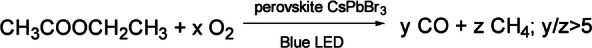




We then systematically studied the CO production of various organic solvents under visible light. As shown in Table 5, in addition to ethyl acetate, we also find that chloroform is a good solvent for visible‐light‐induced photocatalytic CO generation, up to 987 μmol g^−1^ h^−1^ (entries b and c), while most other solvents (i.e., toluene, hexane etc.) do not lead to appreciable CO under the same photocatalytic conditions explored (entries d–g). Interestingly, the moisture in chloroform plays a role in the photocatalytic CO generation, while O_2_, air, CO_2_, or any sparging gas does not impact the CO generation. Furthermore, there is no detectable CH_4_ in this case. In addition to that, we also find that the CsPbBr_3_ NCs have been tuned significantly toward the blue region during photocatalysis, implying formation of CsPbBr_3−*x*_Cl_*x*_. With these factors considered together, the photocatalytic CO generation from CHCl_3_ is clearly different from the case in ethyl acetate, and the mechanism is thus proposed as follows [Eq. 5].

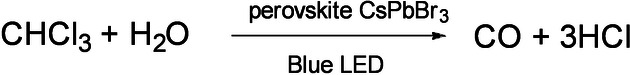




**Table 5:** Solvent exploration for CO and CH_4_.

Entry	Solvent	Condition	Gas	CO	CH_4_
a	CH_3_COOCH_2_CH_3_	CsPbBr_3_ NC	O_2_	151	27
aa	CH_3_COOCH_2_CH_3_	Grinding CsPbBr_3_	O_2_	189	32
ab	CH_3_COOCH_2_CH_3_	Grinding CsPbBr_3_	air	30	6
b	CHCl_3_	NC, 0.1 % v/v H_2_O	N_2_	309	<0.1
ba	CHCl_3_	NC, 0.1 % v/v H_2_O	O_2_	299	<0.1
bb	CHCl_3_	NC, 0.1 % v/v H_2_O	CO_2_	303	<0.1
bc	CHCl_3_	NC, 0.1 % v/v H_2_O and oleylamine	air	987	<0.1
bd	CHCl_3_	Grinding CsPbBr_3_	air	17	<0.1
c	CH_2_Cl_2_	Grinding CsPbBr_3_	air	3	<0.1
d	C_6_H_6_	Grinding CsPbBr_3_	air	<0.1	<0.1
e	MeCN	Grinding CsPbBr_3_	air	<0.1	<0.1
f	Toluene	Grinding CsPbBr_3_	air	<0.1	<0.1
g	Hexanes	Grinding CsPbBr_3_	air	<0.1	<0.1

**Scheme 1:**
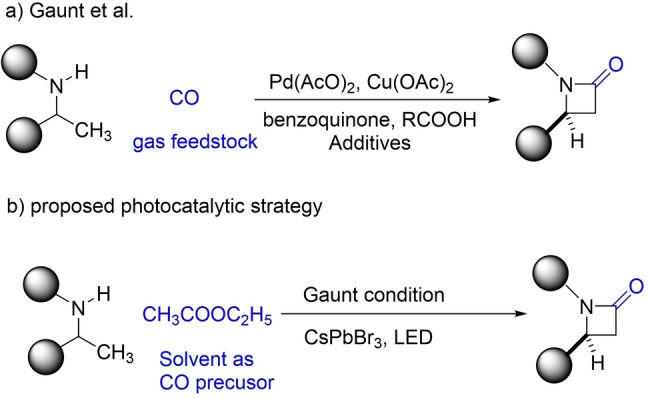
Proposed photocatalytic in situ CO production strategy for β‐lactam synthesis.

**Scheme 2:**
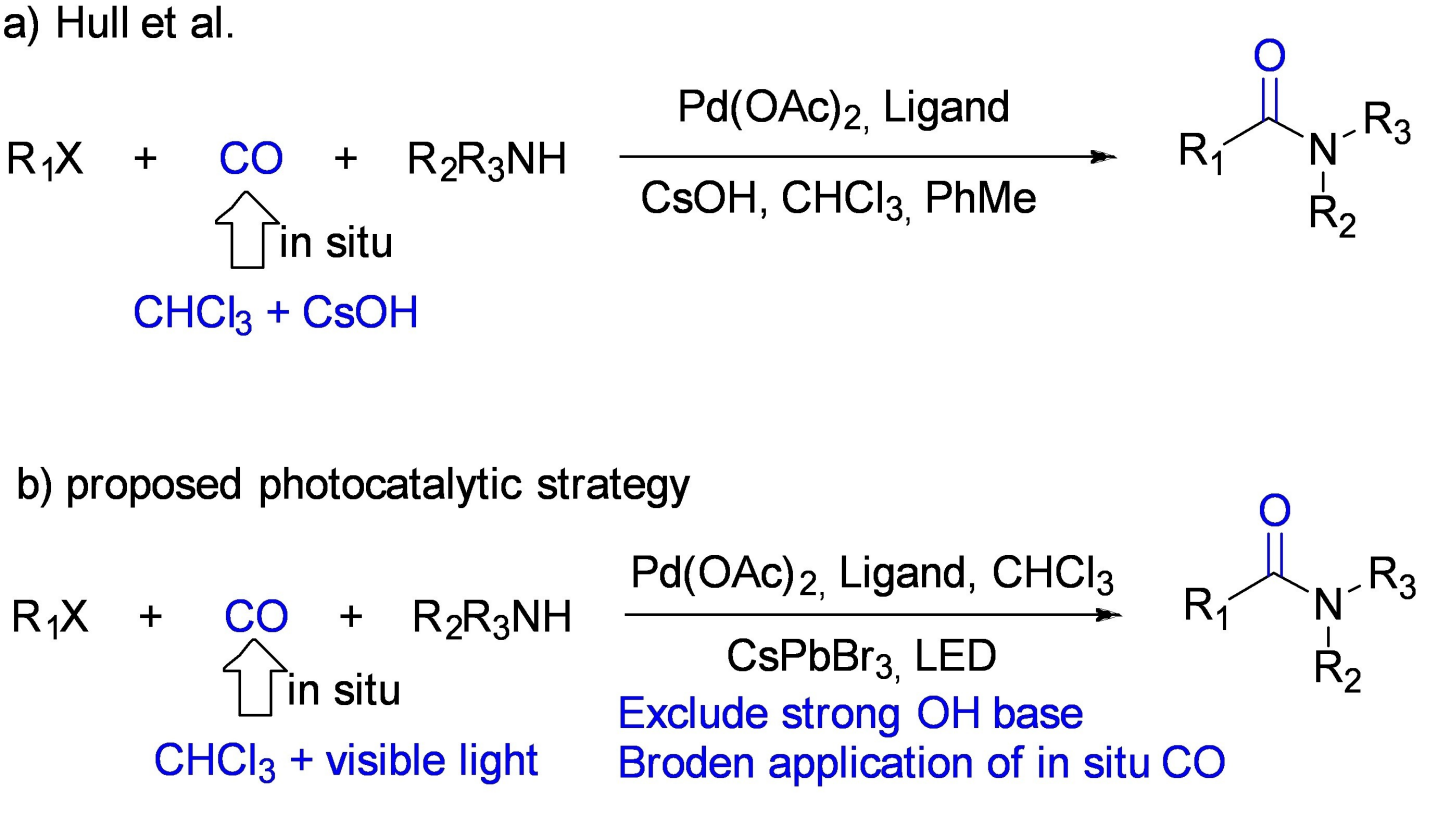
Proposed photocatalytic in situ CO production strategy for aminocarbonylation.

**Potential synthetic application**: From a synthetic point of view, CO is a useful product even if is not from the reduction of CO_2_. It can be dangerous and challenging to handle existing CO infrastructure for organic synthesis; particularly, valuable pharmaceutical synthesis requires a CO feedstock. Under visible‐light photocatalysis, a significant amount of CO can be generated from CHCl_3_ at a rate of ≈1000 μmol g^−1^ h^−1^, rendering a promising strategy to produce in situ CO that may be further employed in challenging synthetic strategies, replacing CO sparging or CO flow as the feedstock. Lately, CO gas has been employed for useful reactions such as pharmaceutical‐related β‐lactam synthesis (Scheme 1).[^50^, ^51^] Hence, we propose a visible‐light‐induced photocatalytic strategy with CHCl_3_ or ethyl acetate as the solvent to replace the toxic CO sparging. Chloroform as a CO surrogate for organic synthesis has been explored previously, but the conversion from CHCl_3_ to CO usually requires an extremely strong base, such as CsOH (Scheme 2a).[^52^, ^53^] Such strong basic conditions restrict the application of CHCl_3_ as the CO surrogate because the strong bases would likely react with the catalyst, the additives, or intermediates in the synthetic cycle. In situ generation of CO under visible‐light illumination at a fast rate, may overcome these limitations and broaden scope in catalytic transformations. Preliminary results using CHCl_3_ as solvent and CsPbBr_3_ NCs as the catalyst without any CO sparging, (Scheme 2b) lead to aminocarbonylation (i.e., morpholino(phenyl)methanone) in a yield of ≈39 % (see the Supporting Information). Further work is currently underway in our laboratory.

## Conclusion

It is important to note that we are not able to validate all of the heterostructures using perovskite with other hybrid catalytic components under which an enhanced catalytic activity, or enhanced photocurrent, have been reported. It is not possible to directly compare other such systems to the pure CsPbBr_3_ NC photocatalytic system. Herein, we are not questioning such observations of a higher catalytic activity, or a higher photocurrent, in hybrid systems. It is true that, under proper modification/hybridization, higher charge‐separation efficiency can indeed enhance the respective catalytic activity (i.e., a higher CO generation rate, or larger photocurrent) in perovskite hybrids.[^23^, ^24^, ^25^, ^26^, ^27^, ^28^, ^29^, ^30^, ^31^, ^32^, ^33^, ^34^, ^35^, ^36^, ^37^, ^38^, ^39^, ^40^, ^41^] Instead, we question whether the generated CO or CH_4_ originates from CO_2_ reduction in an organic solvent system. The catalytic reaction might just be a photoredox organic transformation from the respective organic solvent. We also note that Z‐scheme systems using perovskite with TiO_2_ or other semiconductors are indeed able to oxidize water to generate O_2_.^[30]^ This might be the reason for the claimed ^18^O‐label experiment; however, such Z‐scheme studies have not yet changed the mechanism of cathodic CO_2_ reduction. Perhaps perovskites are still much more effective for ethyl acetate/O_2_ activation than CO_2_.

Here we conclude that CsPbBr_3_‐based photocatalytic CO_2_ reduction in an organic solvent is problematic. The CO is generated during the reaction, but does *NOT* come from CO_2_, according to our strict labeling studies, band energy analysis, and multiple control experiments. The observed CO clearly results from a photocatalytic organic transformation under visible light. Our results corroborate the report that liquid‐phase photocatalysis under full arc or the visible region did not lead to the detection of O_2_ from water nor the carbonaceous product originating from CO_2_ reduction as recently claimed.^[44]^ Note, we do not have the resources to conclude the perovskite photocatalytic CO_2_ reduction in a water vapor environment. It is imperative to clarify whether highly efficient perovskite materials are truly active toward converting solar energy to address the key CO_2_ related issues. Our studies show that such efforts to generate solar fuel from CO_2_ in respective organic solvents may not be successful. Instead, we found that CO generation is exceptionally fast due to the perovskite's strong photocatalytic activity toward activation of ethyl acetate/O_2_ or CHCl_3_/H_2_O, with an incredible CO generation rate up to ≈1000 μmol g^−1^ h^−1^. We also preliminarily employed in situ fast‐generated CO directly from organic solvent under visible‐light illumination for useful organic synthesis, such as pharmaceutically useful photocatalytic aminocarbonylation using a proper organic solvent as a CO surrogate.

## Conflict of interest

The authors declare no conflict of interest.

1

## Supporting information

As a service to our authors and readers, this journal provides supporting information supplied by the authors. Such materials are peer reviewed and may be re‐organized for online delivery, but are not copy‐edited or typeset. Technical support issues arising from supporting information (other than missing files) should be addressed to the authors.

Supporting Information

## Data Availability

The data that support the findings of this study are available in the supplementary material of this article.
