# Supplementary material for: Perovskite Photocatalytic CO2 Reduction or Photoredox Organic Transformation?
Source: Angew Chem Int Ed Engl. 2022 Aug 23;61(39):e202205572. doi: 10.1002/anie.202205572 (PMC9804990; doi:10.1002/anie.202205572)
Supplement: Supplementary file 1 — Supporting Information [file ANIE-61-0-s001.pdf]

Angewandte Chemie
Eine Zeitschrift der Gesellschaft Deutscher Chemiker

Supporting Information

Perovskite Photocatalytic CO_2 Reduction or Photoredox Organic Transformation?

*J. San Martin, N. Dang, E. Raulerson, M. C. Beard, J. Hartenberger, Y. Yan**

Supporting Information

Table of Contents

1. General materials and instruments 2
2. Perovskite $CsPbBr_3$ NC synthesis 2
3. Grinding bulk perovskite $CsPbBr_3$ synthesis 2
4. Characterization 2-5
5. Gas chromatography measurement 5-6
6. Calculation gas rate, μmol/g/h 6
7. Perovskite photocatalytic experimental setup 6-7
8. Band tuning of perovskite for photocatalytic CO generation 7
9. Additional Control experiments. 7
10. Label experiments 7-9
11. Photoluminescence quenching experiments 9-10
12. Summary of Reported $^{13}CO_2$ label experiments (Table S1) 10-11
13. Chloroform as CO surrogate for Organic Synthesis 11-12

1. General materials and instruments

All commercially available reagents and solvents used in this study were purchased from TCI, Fisher or Sigma Aldrich and used without further purification. The labeled solvent was purchased from Cambridge Isotope Laboratories (CIL). Labeled CO_2 gas was from Sigma-Aldrich. Respective gas tanks, including ultra-pure CO_2, were from Air Gas. Gas Flowmeters, 0.1 To 1 Lpm, Viton Seal were purchased from Grainger.

GC measurements were conducted on Shimadzu GC-2010 Plus Tracera with a barrier discharge ionization detector using He (99.9999%) as a carrier gas. PL lifetime measurements were conducted with a DeltaPro TCSPC Lifetime Fluorometer with a 371 nm excitation wavelength. PL spectra were recorded with a Horiba Jobin Yvon Model FluoroMax-4.

2. Perovskite $CsPbBr_3$ NC synthesis

The NC synthesis procedure is adopted from our previous publications.[1] $CsPbBr_3$ nanocrystals (NCs) were synthesized by modifying the hot injection method previously reported. First, a Cs-oleate solution was prepared by charging a 100 ml 3-neck flask with Cs_2CO_3 (0.16g) along with octadecene (6 mL, ODE) and oleic acid (2.5 mL, OA) and dried for one hour under vacuum at 120^oC. The Cs-oleate solution was then followed by N_2 sparging at 150 ^oC until all of the Cs_2CO_3 dissolved in ODE. In a separate 100 ml 3-neck flask, 10 mL ODE and $PbBr_2$ (0.178 g, 0.486 mmol) are dried for one hour under vacuum at 120 ^oC and subsequently purged with N_2 followed by an injection of both oleylamine (1 mL) and OA (1 mL) after the temperature was raised to 150 ^oC. Once the Pb salts dissolved, the temperature was raised to 180^oC and the prepared Cs-oleate solution (1 mL) was swiftly injected into the reactor. After five seconds, the yellow-green reaction mixture was cooled by an ice bath and subsequently washed with methyl acetate (10 mL). After centrifuging at 9000 rpm for five minutes, a yellow-green precipitate was obtained and was further washed with hexanes (10 mL) prior to centrifugation again. The NCs were then vacuum oven dried overnight. The yield was less than 10%.

3. Grinding bulk perovskite $CsPbBr_3$ synthesis

Ground $CsPbBr_3$ was produced by grinding 1mmol of CsBr with 1 mmol $PbBr_2$ using a mortar pestle. After 30 minutes of firm grinding, ~578 mg of yellow microcrystals were produced with moderate green PL under UV-light. Such bulk perovskite power was annealed in the oven at 130°C to remove any possible organics before applying to the photocatalytic reaction.

4. Characterization

Perovskite $CsPbBr_3$ NCs and grinded bulk $CsPbBr_3$ were characterized according to our previous method. [1] The respective characteristic XRD, absorption, photoluminescence, and TEM images are illustrated below. Hitachi H-7500 transmission electron microscope was utilized to measure the TEM images. A Philips Empyrean X-Ray Diffractometer was employed to measure the powder XRD samples.

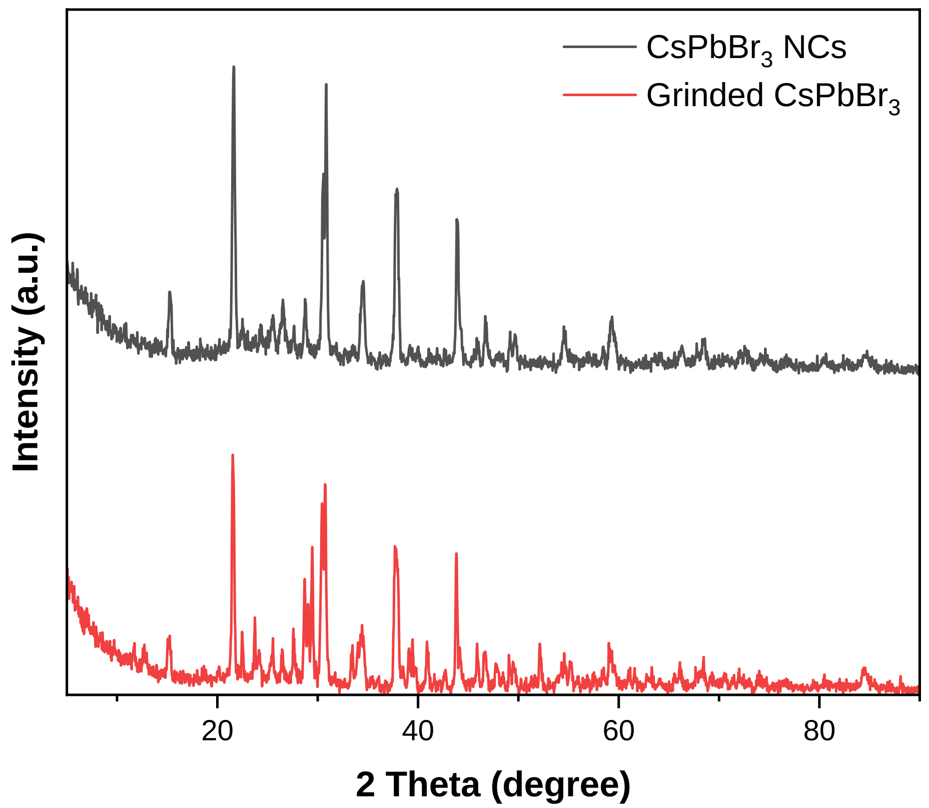

Figure S1: XRD of $CsPbBr_3$ NCs and grinded $CsPbBr_3$.

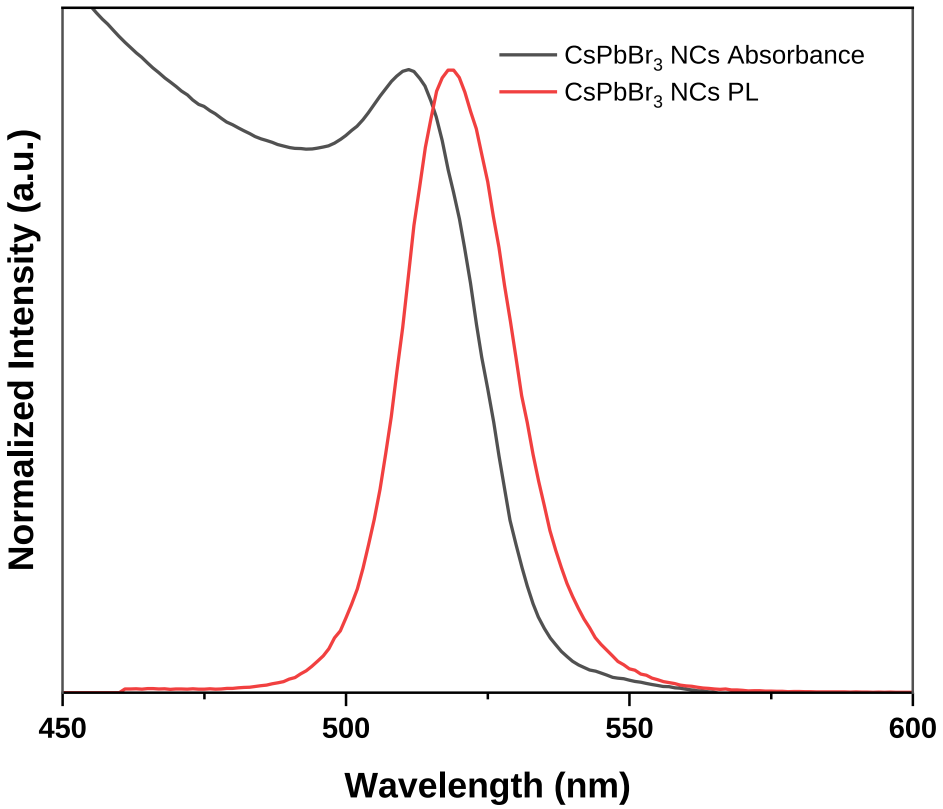

Figure S2: Absorption and PL spectrum of $CsPbBr_3$ NCs. Samples were prepared by suspending 1 mg of material into 3 mL of hexanes. Excitation wavelength used for PL was 420 nm.

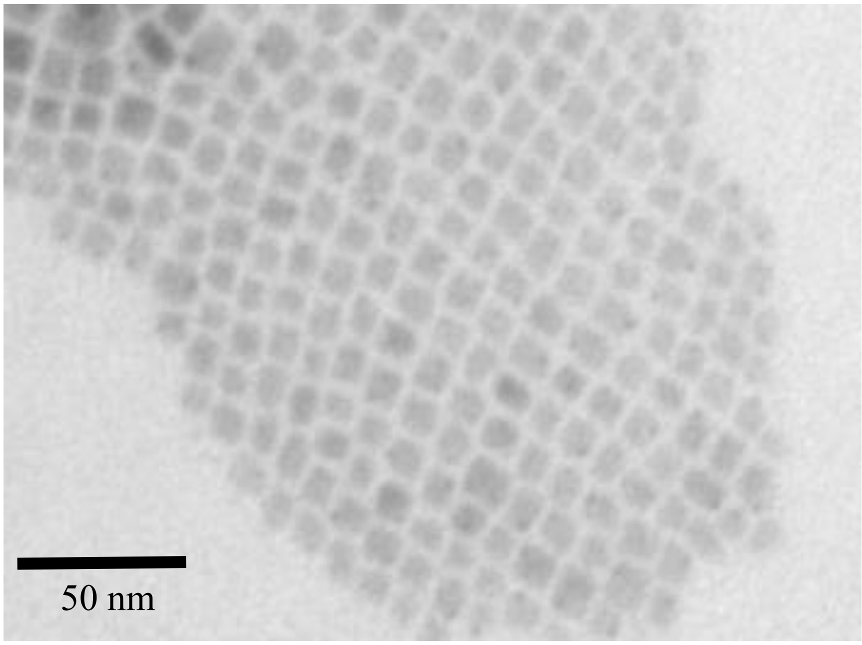

Figure S3: TEM images of $CsPbBr_3$ NCs.

5. Gas chromatography measurement

a) GC calibration:

Calibration Curves were produced for both CO and CH_4 as shown below. (Note: we sincerely acknowledge the Gu Lab at SDSU for highly sensitive GC access)

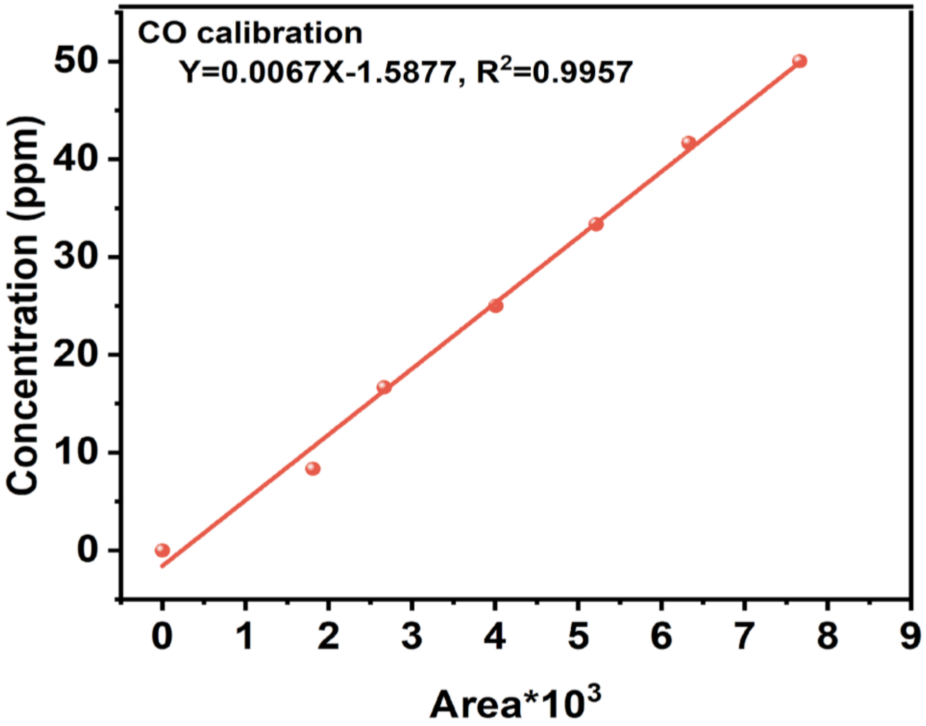

Figure S4: CO calibration curve.

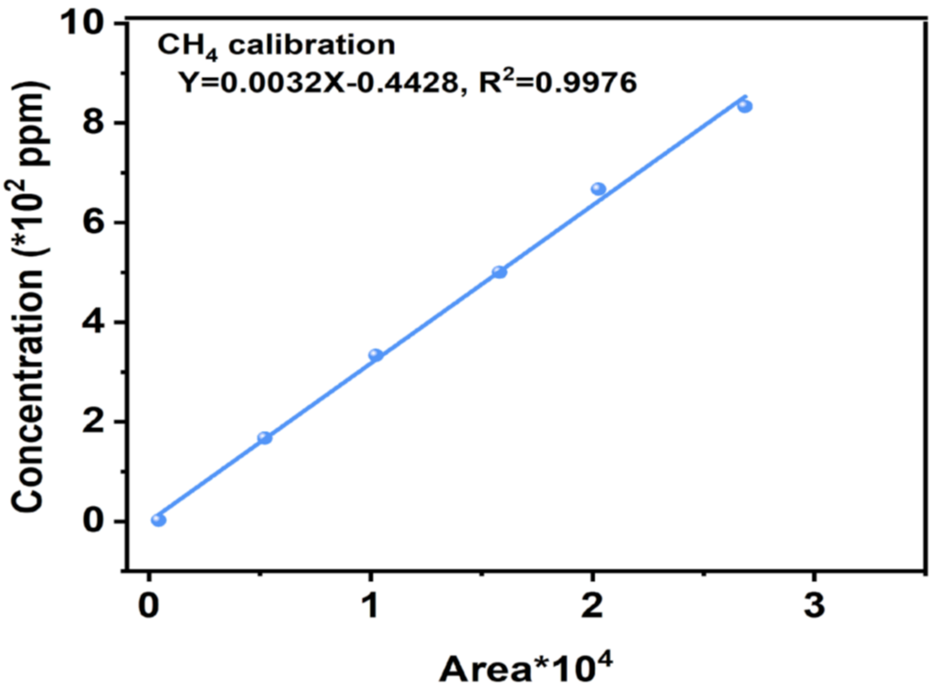

Figure S5: CH_4 calibration curve.

b) Typical GC measurement:

After light irradiation for a desired period of time, a Hamilton gas-tight 50 µL syringe was used to inject 30 uL of the reaction headspace into the GC. The inlet, 0.53mm ID column, and detector were maintained at 200°C, 40°C, and 235°C, respectively. The respective CO or CH_4 amount was determined by the detected respective gas area using the calibration curve above.

6. Calculation of gas rate: umol/g/h

Gas production rate, umol/g/h was determined using the following equation:

$$umol\ g^{-1}h^{-1} = \frac{X\ x\ 10^{-6} x\ 2\ ml\ x \left(\frac{1\ mol}{22400\ ml}\right)}{m\ x\ t}$$

$$where\ X = PPM\ CO\ or\ CH_4,$$

$$t = time\ (h),$$

$$and\ m = mass(g)$$

7. Perovskite photocatalytic experimental setup

A typical photocatalytic experiment has been set up as follows: 1 mg of catalyst is added to a 4 ml vial as well as 2 ml of solvent (i.e., ethyl acetate) containing a stir bar. (Note: 6 uL of water was used if the experiment needed water). Gentle sonication was then used to suspend the catalyst in the solution. The desired gas (i.e., CO_2 or O_2) was then bubbled into the reaction at a flow rate of

~0.1 L/min for 10 minutes to saturate the system. The reaction was then sealed with septum and placed on a stir plate while irradiated by 456 nm Kessil LEDs, intensity (40W).

8. Band tuning of perovskite for photocatalytic CO generation.

Perovskite $CsPbBr_3$ has been tuned with TMSI using our previous method[1], leading to the formation of $CsPbBr_xI_{3-x}$. Highly exchanged $CsPbBr_xI_{3-x}$ NCs with x approaching 3, has also been achieved using largely excess amount of iodide source enabling a VB band close to +1.1 vs. RHE. $CsPbI_3$ NCs were also synthesized to in a similar manner except PbI_2 was used instead of $PbBr_2$.[2] Such perovskite has been employed in photocatalytic reaction set up as follows: 1 mg of catalyst is added to a 4 ml as well as 2 ml of solvent (i.e., ethyl acetate) containing a stir bar. (Note: 6 uL of water was used if the experiment needed water). Gentle sonication was then used to suspend the catalyst in the solution. The desired gas (i.e., CO_2) was then bubbled into the reaction at a flow rate of ~0.1 L/min for 10 minutes to saturate the system. The reaction was then sealed with septum and placed on a stir plate while irradiated by 456 nm Kessil LEDs. After LED illumination, a significant amount of CO has also been observed using this band-tuned perovskite materials with rates reaching up to 2.5 umol/g/h respectively.

9. Additional Control experiments to explore O_2's impact on the photocatalytic outcome.

Air or pure oxygen environment has been employed for control studies with comparison to CO_2, and N_2 atmosphere under the same photocatalytic setup.

In addition, O_2's specific impact has been explored. Before LED illumination, the photocatalytic reaction vial was purged with two needles with two controlled gas flowmeter rates for over 20 minutes. And then the reaction vial was then carefully sealed for photocatalytic illumination. And the headspace gas was detected and measured in the same way as a typical photocatalytic experiment. Note that the CO production rate in this comparison was measured on a 6-hour time scale.

10. Label experiments.

Conditions: 1mL ethyl acetate solvent with 1mg perovskite nanocrystals (~10nm), saturated using various gas for 5 min and sealed with septum, illuminated under 456 nm Kessil LED. Aliquots of the gas from headspace were applied using a gas-tight syringe and detected by GCMS. ^{13}C label using 99 atom% ethyl acetate (From CIL, Cambridge Isotope Laboratory) or 99%atom $^{13}CO_2$ (from Sigma). The details for GCMS data were shown below.

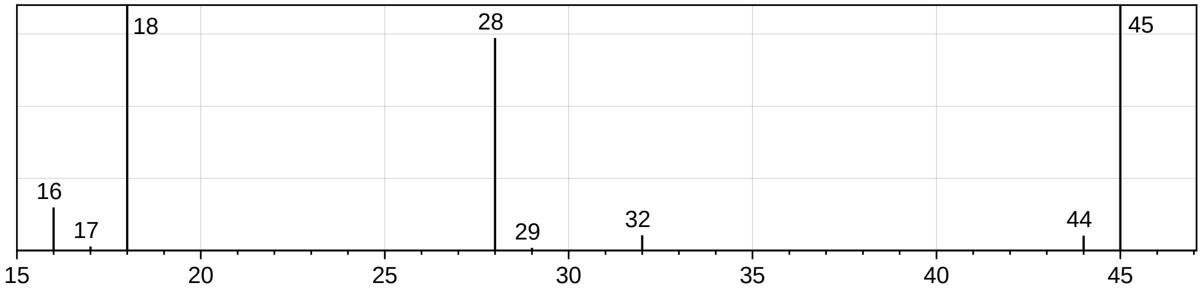

Figure S6: GCMS data of the isotope experiment with $^{13}CO_2$ (m/z=45) sparging into non-labeled ethyl acetate.

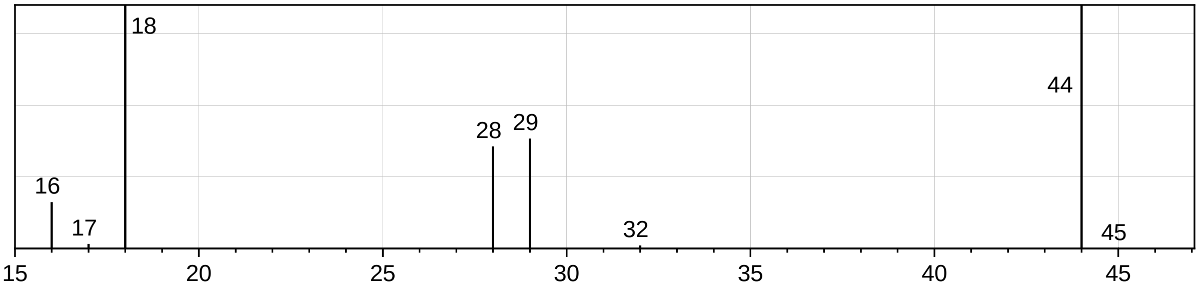

Figure S7: GCMS data of the isotope experiment with $^{12}CO_2$ sparging into 1C-labeled ethyl acetate, $CH_3{}^{13}COOCH_2CH_3$.

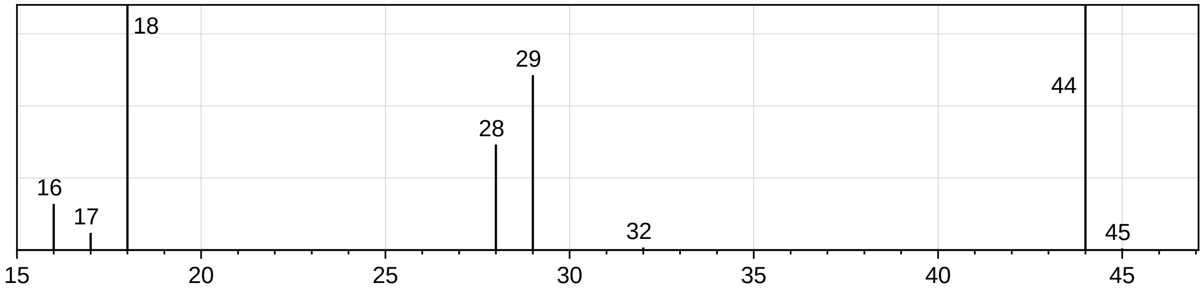

Figure S8: GCMS data of the isotope experiment with $^{12}CO_2$ sparging into 1,2 C-labeled ethyl acetate, $^{13}CH_3{}^{13}COOCH_2CH_3$.

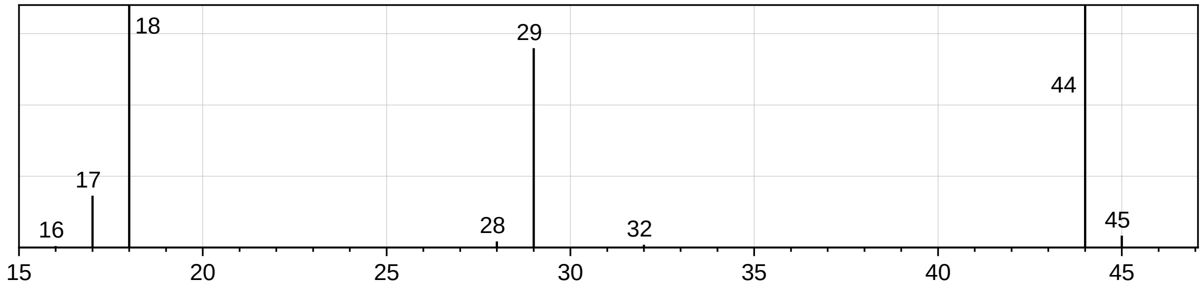

Figure S9: GCMS data of the isotope experiment with $^{12}CO_2$ sparging into 1,2,3,4 C-labeled ethyl acetate, $^{13}CH_3{}^{13}COO^{13}CH_2{}^{13}CH_3$.

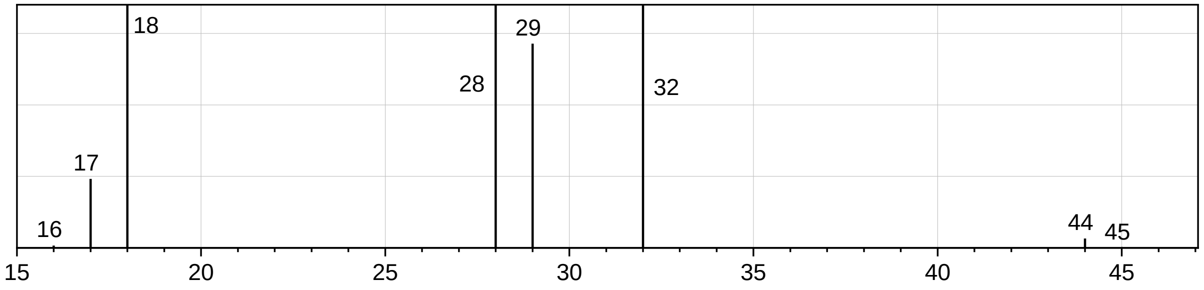

Figure S10: GCMS data of the isotope experiment with air-saturated 1,2,3,4 C-labeled ethyl acetate, $^{13}CH_3{}^{13}COO^{13}CH_2{}^{13}CH_3$. (Note: N_2 and ^{12}CO showing at almost the same position in MS)

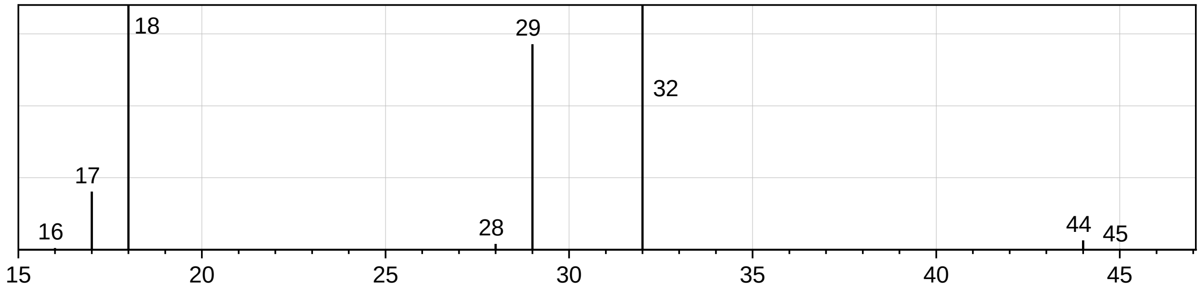

Figure S11: GCMS data of the isotope experiment with O_2-saturated 1,2,3,4 C-labeled ethyl acetate, $^{13}CH_3{}^{13}COO^{13}CH_2{}^{13}CH_3$.

11. Photoluminescence quenching experiments

The samples were prepared by suspending 1 mg of $CsPbBr_3$ NCs in 3mL of hexane. Then, they were saturated using various gases and sealed with septum for further PL measurement. The PL

intensity changes have been collected with a Horiba Jobin Yvon Model under 420nm excitation wavelength. The PL lifetime has been recorded with DeltaPro TCSPC Lifetime Fluorometer FluoroMax-4 with a 371 nm excitation.

12. Summary of Previously reported $^{13}CO_2$ Studies on Perovskite CO_2 Reduction (Table S1)

Catalyst	Products of Reduction	Medium	System	Isotopic Experiment with $^{13}CO_2$ Results	Notes	Ref.
**$CsPbBr_3$ QDs**[3]	CO, CH_4, H_2	Ethyl acetate/water (<0.3 vol %)	PC: 300 W Xe lamp (AM 1.5G)	GCMS: ^{13}CO and $^{13}CH_4$ were both shown with ~100 atom%	No detectable products in the dark or without the catalysts. Under Ar, a certain amount of CO and CO_2 were obtained.	3
**$CsPb(Br_{0.5}/Cl_{0.5})_3$ NCs**[4]	CO, CH_4 (H_2: trace)	ethyl acetate	PC: 300 W Xe lamp (AM 1.5G)	GC-MS: 85% of ^{13}CO comes from $^{13}CO_2$; no information regarding CH4 has been shown even CH4 was reported with appreciable amount	Under Ar, CO yields ~ 1/8 of that under CO_2 (94 µmol/g vs 767 µmol/g)	4
**CsPbBr3 NCs/a-TiO_2**[5]	CO, CH_4, H_2	Ethyl acetate, isopropanol	PC: 150 W Xe lamp (AM 1.5G)	GCMS: signal of ^{13}CO and $^{13}CH_4$ were detected, but not the same atom%	Atom% of ^{13}CO and $^{13}CH_4$ were not the same according to GCMS	5
**$CsPbBr_3$ QDs/g-C_3N_4**[6]	Mainly CO (can also have CH_4 over $CsPbBr_3$ QDs without g-C_3N_4), no H_2	Acetonitrile / water (0.3 vol %)	PC: 300 W Xe lamp (≥ 420 nm)	GCMS: a major signal of ^{13}CO, atom% closed to ~100% was observed,	Under He: insignificant amount of CO;<br>Labeled CH4 was not observed.	6
**$MAPbI_3$ @PCN-221(Fe_x)**[7]	CO, CH_4 (no H_2 or liquid products)	ethyl acetate/water (1.2 vol %)	PC: 300 W Xe lamp (≥ 400 nm)	GCMS: ^{13}CO and $^{13}CH_4$ were shown with ~100 atom%	Under N_2: almost no CO and CH_4 were produced.	7

					GCMS confirmed the oxidation of $H_2{}^{18}O$ to $^{18}O_2$	
**$CsPbBr_3$ @TiO-CN[8]**	CO, CH_4 (very small amount), no other products	Ethyl acetate/water (0.5 vol %)	PC: 300 W Xe lamp (≥ 400 nm)	GCMS: ^{13}CO was shown with ~100 atom%	Experiment with $H_2{}^{18}O$ also showed a major mass signal of $^{18}O_2$	8
**Co@$CsPbBr_3$/Cs_4PbBr_6 NCs[9]**	CO and CH_4	water	PC: 300 W Xe lamp, (100 mW/cm^2, 400 nm)	GCMS: ^{13}CO and $^{13}CH_4$ were shown with ~100 atom%	System displayed no light response without the catalysts and negligible response under N_2.<br>Isotope trace experiment of $H_2{}^{18}O$ obtained a major signal of $^{18}O_2$.	9
**$CsPbBr_3$ -Re (600)[10]**	CO and H_2 (tiny amount)	toluene/ isopropanol (11 vol %)	PC: 150 W Xe lamp (AM 1.5G, 150 mW/cm^2, ≥ 420 nm)	GCMS: ^{13}CO was shown with ~100 atom%<br>No information regarding $^{13}CH_4$	Under N_2, H_2 was detected as the main product with a tiny amount of CO	10
**$CsPbBr_3$ NCs/Pd NS[11]**	CO, CH_4, H_2	Water vapor/ CO_2 gas	PC: 150 W Xe lamp (≥ 420 nm)	GCMS: Signals of $^{13}CH_4$ and ^{13}CO were observed at different atom%	$H_2{}^{18}O$ experiment confirmed water oxidation ($^{18}O_2$)<br>Under N2: CO yield decreases significantly	11
**CsPbBr3 NCs@ZIF-67[12]**	CO, CH_4	Water vapor/ CO_2 gas	PC: 150mW/cm^2 (AM 1.5G)	GCMS: Signals of $^{13}CH_4$ and ^{13}CO were observed at different atom%	$^{18}O_2$ was detected in $H_2{}^{18}O$ experiment.	12

13. Chloroform as a CO surrogate for Morpholino(phenyl)methanone synthesis

Preliminary data for the proposed Scheme-2 in manuscript: orpholino(phenyl)methanone was synthesized by modifying the procedure previously reported.[13] A 4 mL reaction vial containing a stir bar was charged with $CsPbBr_3$ (1 mg), $Pd(OAc)_2$ (1.2 mg), PPh_3 (5.25 mg), iodobenzene (22.4 uL), morpholine (20.8 uL), and chloroform (1.5 mL). The reaction was placed on a stir plate and irradiated by 456 nm Kessil LEDs for 24 hours. After that, it was cooled to room temperature, and the solvent was removed. The crude residue was purified by column chromatography (100:1 dichloromethane:methanol). The detail for the NMR data was shown below. This aligns with the previous report[14]

^{1}H NMR (400 MHz, $CDCl_3$) δ 7.44 – 7.38 (d, J = 3.2 Hz, 5H), 3.89 – 3.38 (m, 8H)

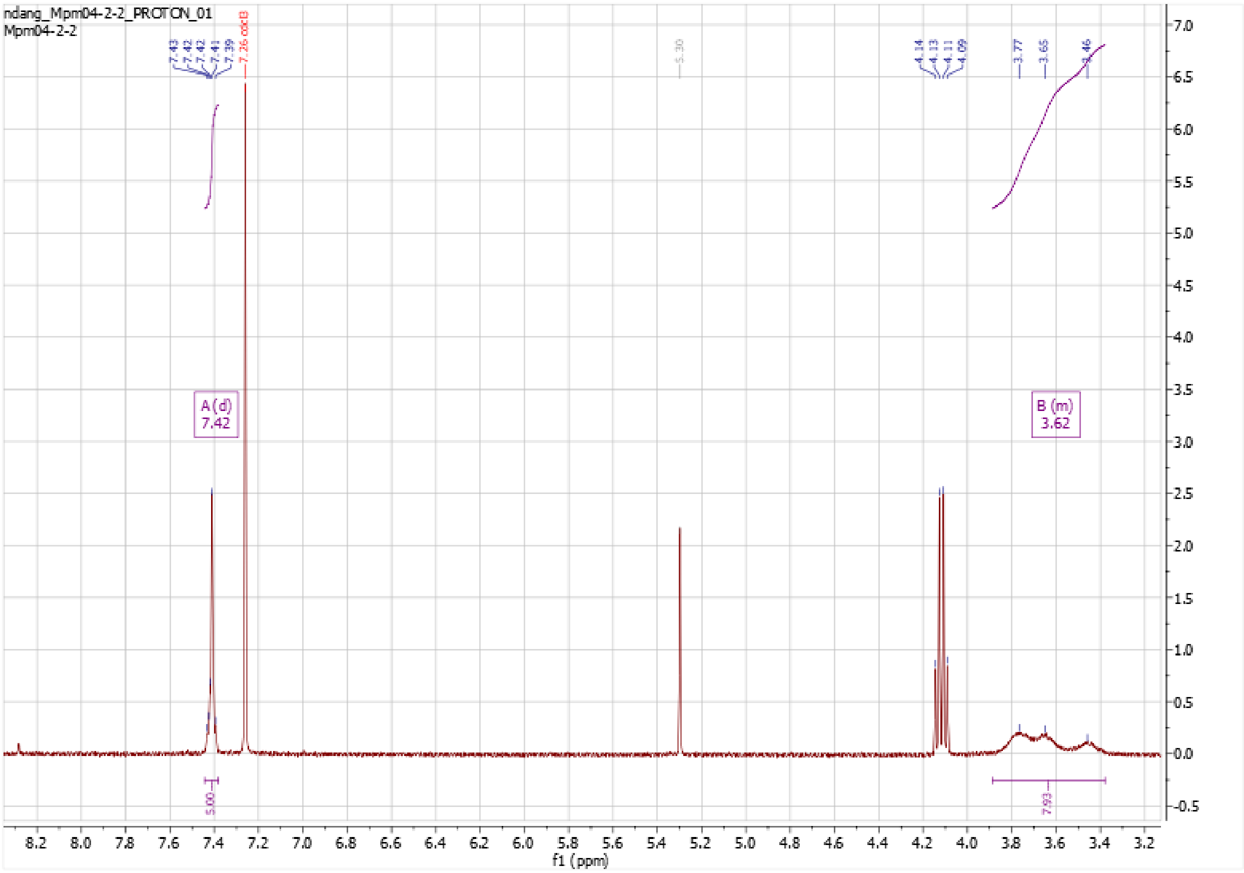

Figure S12: NMR data for morpholino(phenyl)methanone in $CDCl_3$. Peaks at 5.30 and 4.09-4.14 ppm are solvents (DCM and ethyl acetate respectively).

References

[1] X. Zhu, Y. Lin, J. San Martin, Y. Sun, D. Zhu, Y. Yan, *Nat. Commun.* **2019**, *10*, 2843.

[2] A. Swarnkar, A. R. Marshall, E. M. Sanehira, B. D. Chernomordik, D. T. Moore, J. A. Christians, T. Chakrabarti, J. M. Luther, Science **2016**, *354*, 92-95.

[3] J. Hou, S. Cao, Y. Wu, Z. Gao, F. Liang, Y. Sun, Z. Lin, L. Sun, *Chemistry – A European Journal* **2017**, *23*, 9481-9485.

[4] S.-H. Guo, J. Zhou, X. Zhao, C.-Y. Sun, S.-Q. You, X.-L. Wang, Z.-M. Su, *Journal of Catalysis* **2019**, *369*, 201-208.

[5] Y.-F. Xu, X.-D. Wang, J.-F. Liao, B.-X. Chen, H.-Y. Chen, D.-B. Kuang, *Advanced Materials Interfaces* **2018**, *5*, 1801015.

[6] M. Ou, W. Tu, S. Yin, W. Xing, S. Wu, H. Wang, S. Wan, Q. Zhong, R. Xu, *Angewandte Chemie International Edition* **2018**, *57*, 13570-13574.

[7] L.-Y. Wu, Y.-F. Mu, X.-X. Guo, W. Zhang, Z.-M. Zhang, M. Zhang, T.-B. Lu, *Angewandte Chemie International Edition* **2019**, *58*, 9491-9495.

[8] X.-X. Guo, S.-F. Tang, Y.-F. Mu, L.-Y. Wu, G.-X. Dong, M. Zhang, *RSC Advances* **2019**, *9*, 34342-34348.

[9] Y.-F. Mu, W. Zhang, X.-X. Guo, G.-X. Dong, M. Zhang, T.-B. Lu, *ChemSusChem* **2019**, *12*, 4769-4774.

[10] Z.-C. Kong, H.-H. Zhang, J.-F. Liao, Y.-J. Dong, Y. Jiang, H.-Y. Chen, D.-B. Kuang, *Solar RRL* **2020**, *4*, 1900365.
[11] Y.-F. Xu, M.-Z. Yang, H.-Y. Chen, J.-F. Liao, X.-D. Wang, D.-B. Kuang, *ACS Applied Energy Materials* **2018**, *1*, 5083-5089.
[12] Z.-C. Kong, J.-F. Liao, Y.-J. Dong, Y.-F. Xu, H.-Y. Chen, D.-B. Kuang, C.-Y. Su, *ACS Energy Letters* **2018**, *3*, 2656-2662.
[13] S. N. Gockel, K. L. Hull, *Organic Letters* **2015**, *17*, 3236–3239.
[14] T. Ohshima, T. Iwasaki, Y. Maegawa, A. Yoshiyama, K. Mashima, *J Am Chem Soc* **2008**, *130*, 2944–2945.
